# Initial experience with 3D isotropic high-resolution 3 T MR arthrography of the wrist

**DOI:** 10.1186/s12891-016-0890-5

**Published:** 2016-01-16

**Authors:** John K. Sutherland, Taiki Nozaki, Yasuhito Kaneko, Hon J. Yu, Gregory Rafijah, David Hitt, Hiroshi Yoshioka

**Affiliations:** University of California, Irvine, Department of Radiological Sciences, UCI Medical Center 101 The City Dr. South, Route 140, Orange, CA 92686 USA; University of California, Irvine, Department of Radiological Sciences and John Tu and Thomas Yuen Center for Functional Onco-Imaging Irvine, Irvine, CA USA; University of California, Irvine, Department of Orthopaedic Surgery, Irvine, USA; Philips Medical Systems, Cleveland, OH USA

**Keywords:** Musculoskeletal MRI, 3D MR imaging, MR arthrography, Wrist MRI

## Abstract

**Background:**

Our study was performed to evaluate the image quality of 3 T MR wrist arthrograms with attention to ulnar wrist structures, comparing image quality of isotropic 3D proton density fat suppressed turbo spin echo (PDFS TSE) sequence versus standard 2D 3 T sequences as well as comparison with 1.5 T MR arthrograms.

**Methods:**

Eleven consecutive 3 T MR wrist arthrograms were performed and the following sequences evaluated: 3D isotropic PDFS, repetition time/echo time (TR/TE) 1400/28.3 ms, voxel size 0.35x0.35x0.35 mm, acquisition time 5 min; 2D coronal sequences with slice thickness 2 mm: T1 fat suppressed turbo spin echo (T1FS TSE) (TR/TE 600/20 ms); proton density (PD) TSE (TR/TE 3499/27 ms). A 1.5 T group of 18 studies with standard sequences were evaluated for comparison. All MR imaging followed fluoroscopically guided intra-articular injection of dilute gadolinium contrast. Qualitative assessment related to delineation of anatomic structures between 1.5 T and 3 T MR arthrograms was carried out using Mann–Whitney test and the differences in delineation of anatomic structures among each sequence in 3 T group were analyzed with Wilcoxon signed-rank test. Quantitative assessment of mean relative signal intensity (SI) and relative contrast measurements was performed using Wilcoxon signed-rank test.

**Results:**

Mean qualitative scores for 3 T sequences were significantly higher than 1.5 T (*p* < 0.01), with isotropic 3D PDFS sequence having highest mean qualitative scores (*p* < 0.05). Quantitative analysis demonstrated no significant difference in relative signal intensity among the 3 T sequences. Significant differences were found in relative contrast between fluid-bone and fluid-fat comparing 3D and 2D PDFS (*p* < 0.01).

**Conclusions:**

3D isotropic PDFS sequence showed promise in both qualitative and quantitative assessment, suggesting this may be useful for MR wrist arthrograms at 3 T. Primary reasons for diagnostic potential include the ability to make reformations in any obliquity to follow the components of ulnar side wrist structures including triangular fibrocartilage complex. Additionally, isotropic imaging provides thinner slice thickness with less partial volume averaging allowing for identification of subtle injuries.

## Background

The wrist, given its relative small size compared to other joints and complex arrangement of ligaments, requires high-resolution, high signal-to-noise, and small field of view MR imaging for accurate assessment of anatomy and pathology. When attention is required at the ulnar side of the wrist for evaluation of triangular fibrocartilage complex (TFCC) or capsular injury, direct MR arthrography has become an increasingly utilized modality, allowing for improved visualization of the closely grouped ligamentous structures following distention of the joint with injected contrast [1–3]. In addition to joint distension, intra-articular contrast allows for visualization of contrast leakage/extravasation and has been shown to be particularly useful in evaluating partial ligamentous tears including of the TFCC [4]. With increasingly widespread use of 3 T MR in musculoskeletal imaging, the wrist included, 3 T MR has been shown to be sensitive and specific for wrist ligament tears in addition to improved visualization of normal anatomy [5, 6]. The addition of 3D isotropic imaging has also shown utility in musculoskeletal imaging, including of the knee [7, 8]. More recent literature involving 3 T 3D isotropic imaging of the wrist in healthy volunteers has been carried out, highlighting the advantages of multi-planar reconstructions and additional post-processing capabilities afforded by isotropic imaging which prove useful in evaluating the intricate anatomy of the wrist [9].

Our study was performed to evaluate image quality of 3 T MR arthrograms which include 3D isotropic proton density weighted fat suppressed sequence (PDFS). Comparison was carried out with standard 1.5 T MR arthrogram studies and additional quantitative analysis of relative signal intensity and relative contrast of the individual 3 T sequences was also performed.

## Methods

Retrospective review of MR images was carried out following University of California, Irvine institutional review board approval. Given our study was a retrospective review, informed consent was waived by the IRB. For the 3 T group, there were 11 total patients (ages 17 to 58 years, average age 33 years; four females and seven males; nine right and two left wrist) collected from studies performed from 11/2012 to 2/2015. Ten of the 11 included 3D isotropic PDFS sequence. 3 T sequence imaging parameters can be found in Table 1. All 3 T images were acquired on a Phillips 3 T system using 8 channel wrist coil (Achieva, Philips HealthcareVR, Best, The Netherlands). A parallel imaging technique called sensitivity encoding (SENSE) was used both in the 2D and 3D sequences at 3 T. All 3D images were obtained with the driven equilibrium (DRIVE) technique. The 3D isotropic PD FSE sequence uses flip angle sweep, short and nonvolume selective refocusing pulse, and SENSE, making it possible to get shorter echo spacing and a better signal-to-noise ratio [10].

**Table 1:** Imaging parameters of 3 T MR arthrogram

Mode	3D	2D	2D	2D
Sequence	Cor PD	Cor T1	Cor PD	Cor PD
Fat saturation	SPIR	SPIR	SPIR	None
Image matrix	200x200	200x198, 240x168	268x250	392x284, 296x234
Slices	151	20–22	20	20
FOV (mm)	70	70	70	70
Slice thickness (mm)	0.35	2	2	2
Slicegap (mm)	0	0.2	0.2	0.2
TE (ms)	28.3	20	27	27–30
TR (ms)	1400	600	3499–4083	3181–3500
BW (Hz/pixel)	179–417	169–179	184–207	184–207
Echo train length	70–88	3	13	13
NEX	2	1	3	2
Parallel imaging	SENSE	SENSE	SENSE	SENSE
Acquisition time (min)	5–5.7	3.5	6	2.2–3.8

For the 1.5 T group there were 18 total patients (ages 15 to 58 years, average age 31 years, six females and 12 males, eight right and eight left wrists) from review of MR arthrograms from 2006 through 2013. 1.5 T imaging parameters can be found in Table 2. All images in this group were acquired on Siemens 1.5 T system (Avanto, Siemens AG, Berlin, Gemany).

**Table 2:** Imaging parameters of 1.5 T MR arthrogram

Mode	2D	2D	2D
Sequence	Cor T1	Cor T1 FS	Cor SPGR MEDIC *N* = 11
Fat saturation	None	Yes	None
Image matrix	256 x 256	256 x 256	256 x 256
Number of slices	12–15	11–17	12–14
FOV (mm)	80–120	75–120	80–120
Slice thickness (mm)	3	3	3
Slicegap (mm)	0.27–0.6	0.27–0.6	0.27
TE (ms)	15–24	8–15	22
TR (ms)	480–681	400–550	719
TI	N/A	N/A	130
Flip angle	N/A	N/A	30
BW (Hz/pixel)	65–140	80–140	195
Echo train length	1–3	1–3	1
NEX	1	2	1

All studies of both 1.5 T and 3 T groups were obtained following injection of 3–4 ml of dilute gadolinium contrast mixture (15 mL sterile saline, 5 mL iodinated contrast, and 0.1 mL of gadolinium contrast) into the radiocarpal joint under fluoroscopy using standard department protocol. The patients were imaged within 30 min of injection.

### Qualitative analysis

A total of 11 wrists from the 3 T group and 18 from the 1.5 T group were independently evaluated on AGFA Impax PACS workstation (AGFA Morsel, Belgium) by two experienced musculoskeletal radiologists (HY (R1), 26 years of experience and TN (R2), 13 years of experience) without knowing clinical data. For evaluation of the 3 T group, each of the 3 imaging sequences (T1FS, PDFS, 3D isotropic PDFS) were graded separately on the delineation of anatomic structures and quality for detection of pathology including presence of contrast leak, presence/absence of abnormality in the prestyloid recess, ability to visualize triangular ligament styloid attachment and presence/absence of any associated injury, status of remaining TFCC, and ability to visualize ulnar collateral ligament attachment and presence/absence of any related injury. For the above pathology except contrast leak, each 3 T sequence (T1FS, PDFS, 3D isotropic PDFS) image quality was graded from 0 to 4; 0 for no visualization, 1 for poor visualization, 2 satisfactory, 3 good, and 4 excellent. Contrast leak was classified as major or minor, with major leak being a contrast leakage outside the joint capsule with signal intensity approximately equal to signal intensity of the injected contrast within the radiocarpal joint and minor leak being less signal intensity than injected contrast. Confidence level of leak detection was graded from 0 to 3 with 0 for no leak, 1 possible, 2 probable, and 3 definite leak. For UCL attachment and injury, a fourth sequence was also evaluated (PD without fat suppression) because ligamentous attachment is generally better identified without fat suppression. One study was a post operative study from prior repair of TFCC injury and given post surgical changes, the anatomic evaluation was unable to be accurately performed, and only overall image quality of the sequences was evaluated. For the 18 wrist studies in the 1.5 T group, the same anatomic and pathologic structures were evaluated in a similar manner but instead of assigning a score from 0 to 4 for each individual sequence, an overall score for the study was assigned using the best image quality from the 3 sequences evaluated (T1, T1FS, and Multi-Echo Data Image Combination (MEDIC)).

### Quantitative analysis

**Fig. 1:**
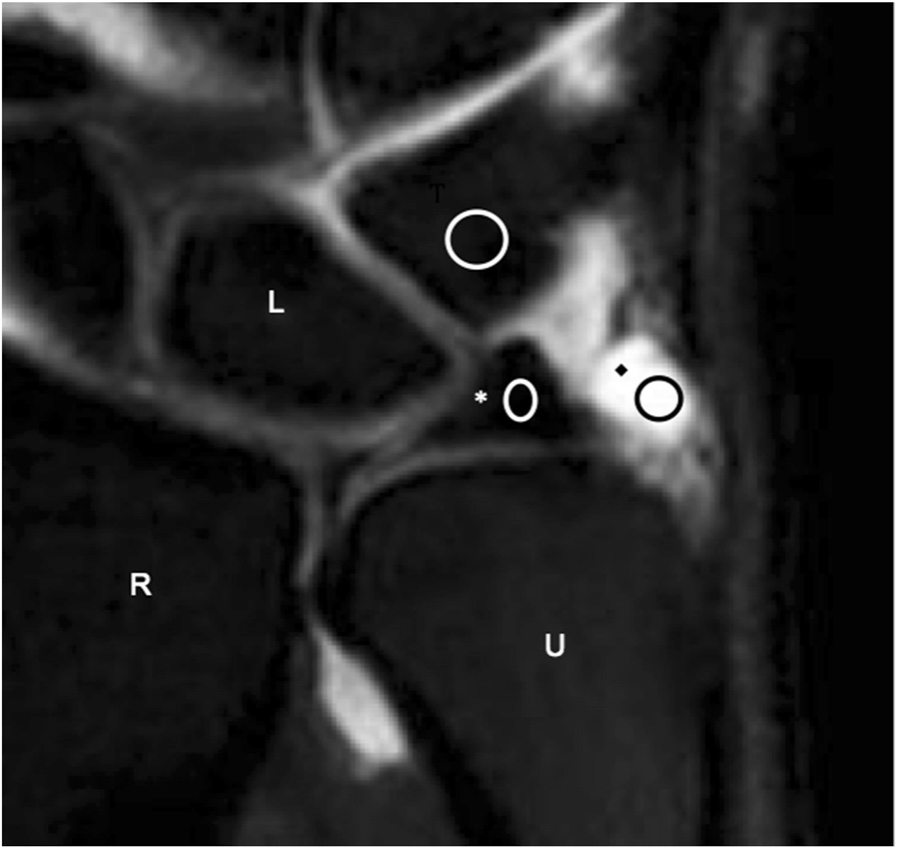
3 T 3D PDFS image with marks indicating region of interest (ROI) measurements used in quantitative analysis. Average signal intensity of the contents of the ellipse was measured (R-radius, U-ulna, *-TFCC disc, ♦-prestyloid recess)

Quantitative analysis was performed for the 11 3 T studies. For each of the 3 T wrist sequences (2D T1FS, 2D PDFS, isotropic 3D PDFS), ROIs for each patient were drawn and relative signal intensity (SI) and relative contrast were measured using. ROI’s were identical in size and placed in identical positions on matching sections. Fluid was measured from the space adjacent to TFCC, prestyloid recess, or intercarpal space (Fig. 1). The mean and standard deviation (SD) of SI were determined for the disc of the TFCC, fluid, cartilage, and bone marrow. Then, relative SI and relative contrast were used for direct comparison of image quality between the 2D FSE and 3D isotropic FSE MR images both with and without fat suppression, because all sequences in this study were obtained with a parallel imaging technique. Relative SI of each structure was calculated as SI/SD, and relative contrast of structure A (a) to structure B (b) was calculated as (SI_a_-SI_b_) / (SD_a_^2^ + SD_b_^2^)^1/2^ [9, 11].

### Statistical analysis

In the qualitative assessments, differences in the delineation of anatomic structures between 1.5 T and 3 T MR arthrography were statistically analyzed using the Mann–Whitney test, and differences in the delineation of anatomic structures among each sequence (2D T1FS, 2D PDFS, isotropic 3D PDFS and 2D PD) analyzed using the Wilcoxon signed-rank test. Inter-rater reliability was measured with percentage of exact agreement and agreement within a range of +/−1 score point [12]. Cohen’s kappa was also calculated to assess inter-rater reliability for the presence or absence of each anatomical structure’s injury. The following ratings for the interpretation of kappa were used; poor (<0.40), moderate (0.40–0.59), good (0.60–0.80), and excellent (>0.80). In the quantitative assessments, comparison of mean relative SI and relative contrast between 3 T 2D FSE and 3 T 3D isotropic FSE MR images were performed using the Wilcoxon signed-rank test. *P* values of less than 0.05 were considered to indicate a significant difference. All statistical analyses were performed using R version 3.0.2 for windows software (R Development Core Team, Vienna, Austria).

## Results

### Qualitative analysis

**Fig. 2:**
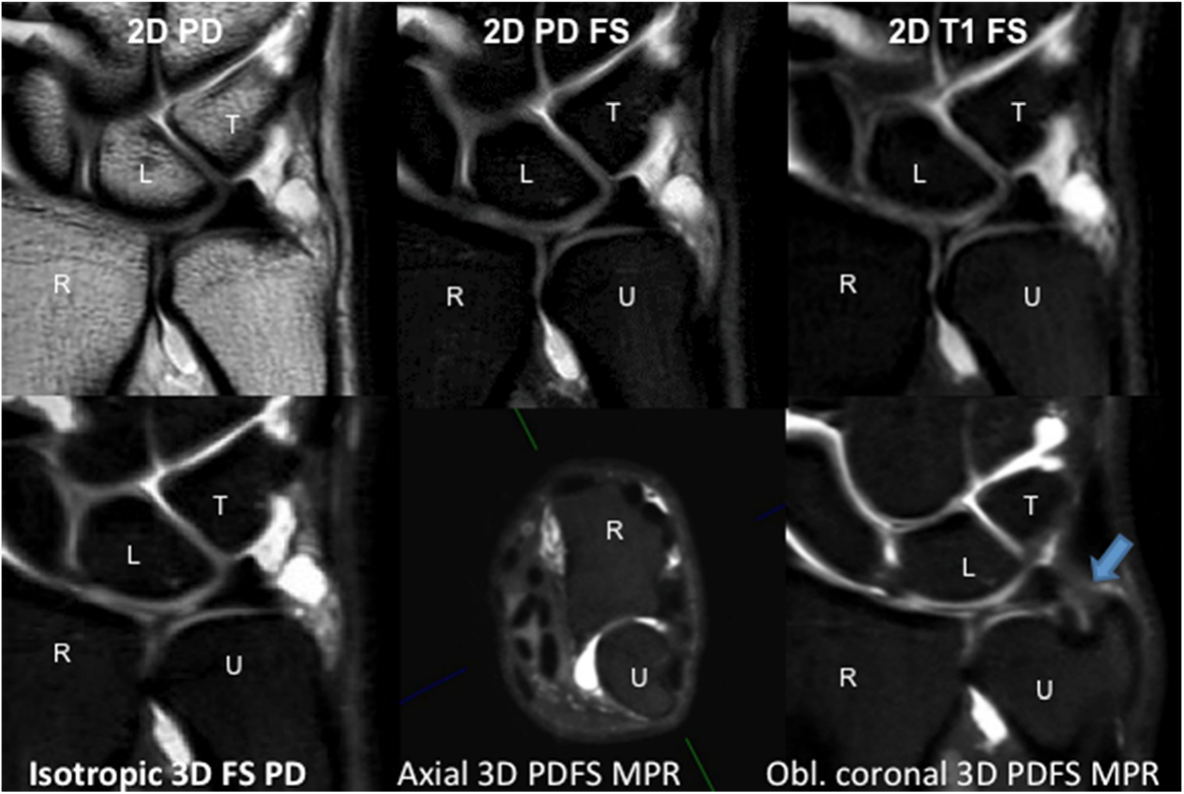
Selected images from 3 T MR arthrogram at the same slice. Bottom right *blue arrow* shows styloid attachment of TFCC, not identified on other sequences due to image plane and slice thickness. *Green line* on axial 3D PDFS MPR image indicates direction of oblique coronal plane. (R-radius, U-ulna, L-lunate, T-triquetrum)

**Fig. 3:**
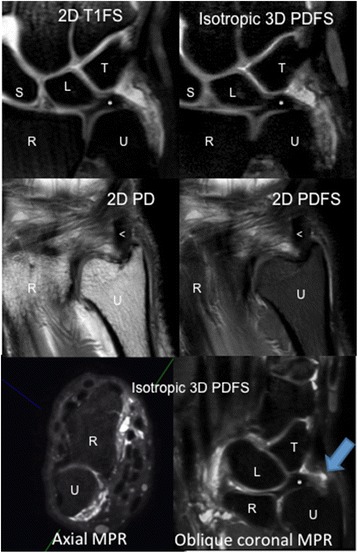
Images from 3 T study. *Top* images are showing that at the level of the disc of TFCC, the ulnar styloid is out of plane. Middle images show ulnar styloid with TFCC disc out of plane. Bottom images: axial, left, from which oblique multi-planar reconstruction (MPR), right, was made (with *green line* in bottom left image indicating direction of oblique coronal plane). *Blue arrow* showing wavy fibers and focal defect compatible with partial tear of the distal lamina of the triangular ligament, which is not seen with standard coronal imaging due to orientation of the ligament and slice selection. (R-radius, U-ulna, S-scaphoid, L-lunate, T-triquetrum, <−extensor carpi ulnaris in middle images)

Mean values of qualitative analysis calculated from individual scores are presented in Table 3. Each 3 T sequence mean value was statistically significantly higher than that for overall image quality of 1.5 T studies (see Table 3). Within the 3 T group, the 3D sequence had the highest average scores for the five separate categories (contrast leak, prestyloid recess abnormality, styloid attachment of TFC, remaining TFC, and UCL), and the 3D isotropic sequence was statistically significantly superior in the image quality of the triangular ligament compared with the T1FS (*p* = 0.047) and PDFS (*p* = 0.026). Figure 2 shows examples 3 T coronal MR arthrogram images as well as oblique coronal MPR image created from 3D isotropic PDFS sequence. Oblique coronal MPR clearly demonstrates ulnar attachment of the triangular ligament of the TFCC. In Fig. 3, only oblique coronal MPR is able to demonstrate a focal partial tear of the distal lamina of the triangular ligament. At the level of the disc of the TFCC, the ulnar styloid is not included in the coronal plane (see the top row in Fig. 3) and at the level of ulnar styloid, the TFCC is not shown in the coronal plane (see the middle row in Fig. 3). This happened very frequently in a true coronal plane because the transverse axis of the radius, which is parallel to the coronal plane, is not the identical to the transverse axis of the ulnar head (for example oblique axial image in Figs. 2 and 3).

**Table 3:** Mean scores for qualitative analysis of MR arthrogram of the wrist

		Contrast leak	Prestyloid recess	Triangular ligament	Remaining TFCC	UCL
3 T MRI	2D FST1	3.8	3.5	3.2	3.7	3.3
	2D FSPD	3.6	3.5	3.1	3.6	3.4
	Isotropic 3D FSPD	3.9	3.9	3.9	3.8	3.8
	2D PD	ー	ー	ー	ー	3.6
1.5 T MRI	T1 or FST1 or MEDIC	2.7	2.5	2.1	2.6	2.2

1.5 T vs 3 T: for contrast leak, all *p* < 0.001; for prestyloid recess, 1.5 T vs 3D (*p* < 0.001), 1.5 T vs FST1 (*p* = 0.003), 1.5 T vs FSPD (*p* = 0.003); for triangular ligament, 1.5 T vs 3D (*p* < 0.001), 1.5 T vs FST1 (*p* = 0.001), 1.5 T vs FSPD (*p* = 0.002); for remaining TFCC, all *p* < 0.001; for UCL, 1.5 T vs FST1 (*p* = 0.001), others *p* < 0.001

Among 3 T sequences: 3D vs FST1 (*p* = 0.047), 3D vs FSPD (*p* = 0.026) regarding evaluation of triangular ligament

Contrast leak was identified in 10 of 11 3 T studies; 4 classified as major and 6 as minor leak with higher confidence than 1.5 T, (Table 4). For 1.5 T studies, 12 of 18 showed contrast leak with 4 major and 8 minor.

**Table 4:** Evaluation of contrast leak

3 T	Contrast leak	Major (M) or minor (m)	1.5 T	Contrast leak	Major (M) or minor (m)
1	1	m	1	2	m
2	3	m	2	3	M
3	0	N/A	3	0	N/A
4	3	M	4	0	N/A
5	3	M	5	0	N/A
6	3	m	6	0	N/A
7	3	M	7	2	m
8	3	m	8	2	m
9	3	m	9	0	N/A
10	3	M	10	3	m
11	3	m	11	1	m
			12	3	M
			13	0	N/A
	Contrast Leak:		14	2	m
	3: definite		15	3	m
	2: probable		16	3	m
	1: possible		17	3	M
	0: no leak		18	3	M

Major leak (M): contrast leakage outside joint capsule with signal intensity equal to that of injected contrast

Minor leak (m): contrast leakage with signal intensity less than injected contrast signal intensity

The results of inter-rater (R1-R2) agreement for each evaluation are shown in Table 5. All sequences at 3 T and 1.5 T demonstrated high inter-rater agreement within one point (90.9–100 at 3 T and 94.4–100 % at 1.5 T). Inter-rater agreement of the presence or absence of each anatomical structure’s injury was excellent (k = 0.83–1.00) on 3.0 T-MR arthrography, and good or excellent (k = 0.71–0.89) on 1.5 T-MR arthrography. 3 T-MR arthrography was superior compared with 1.5 T-MR arthrography on the evaluation of each anatomical structure’s injury.

**Table 5:** Inter-rater agreement (quantitative and qualitative analysis)

	Percentage of inter-rater (R1-R2) agreement(%) exact/within 1 point					Cohen’s kappa value	
	3 T-MRI				1.5 T-MRI		
	2D FST1	2D FSPD	Isotropic 3D FSPD	2D PD	T1 or FST1 or MEDIC	3 T-MRI	1.5 T-MRI
Contrast leak	36.4/100	54.5/100	100/100	N/A	72.2/100	1.00	0.89
Prestyloid recess	36.4/100	90.9/100	90/100	N/A	77.8/100	1.00	0.68
Triangular ligament	45.5/90.9	45.5/90.9	90/100	N/A	38.9/100	0.86	0.81
Remaining TFCC	45.5/90.9	63.6/90.9	80/100	N/A	61.1/94.4	0.86	0.80
UCL	54.5/90.9	63.6/90.9	70/100	72.7/100	55.6/94.4	0.83	0.71

### Quantitative analysis

Mean values for quantitative measurements of relative signal intensity of fluid, bone marrow, TFCC, and fat as well as relative contrast of fluid to bone, fluid to TFCC, and fluid to fat are shown in Table 6. There were no significant differences in relative SI between the three evaluated 3 T sequences, however the 3D isotropic PDFS sequence showed significantly higher relative contrast of fluid to bone and fluid to fat compared to the 2D PDFS sequence (*p* < 0.01). The 2D T1FS sequence also showed significantly higher relative contrast of fluid to fat compared to 2D PDFS (*p* < 0.05).

**Table 6:** Quantitative analysis of high-resolution MR arthrogram at 3 T

		2D FST1 (mean ± SD)			2D FSPD			Isotropic 3D FSPD		
Relative SI	Fluid	106.59	±	104.16	49.56	±	25.87	75.93	±	67.01
	Bone	6.17	±	8.57	7.61	±	6.75	4.94	±	3.98
	TFCC	2.88	±	3.94	5.39	±	6.58	4.54	±	3.92
	Fat	11.36	±	9.42	10.18	±	5.06	9.03	±	7.28
Relative contrast	Fluid-bone	**57.02**	**±**	**48.77**	25.80	±	10.45	**47.19**	**±**	**19.14**
	Fluid-TFCC	42.52	±	21.65	39.19	±	21.21	55.39	±	48.94
	Fluid-fat	**43.54**	**±**	**24.31**	24.59	±	12.63	**42.85**	**±**	**18.75**

Bolded are statistically significant in relation to 2D FSPD

Fluid-bone: FST1 vs FSPD, *p* = 0.037 and 3D vs FSPD, *p* = 0.005

Fluid-fat: FST1 vs FSPD, *p* = 0.022 and 3D vs FSPD, *p* = 0.007

## Discussion

Evaluation of the ulnar side of the wrist remains a challenge in everyday medical practice, particularly due to the complex nature of triangular fibrocartilage complex. This is complicated by discussion involving anatomic nature of ulnar wrist structures including ulnomeniscal homologue [13–17] and ulnar collateral ligament complex [18–21], both of which have been debated in the literature. Despite the nuances in the anatomy, direct MR arthrography with injected intra-articular gadolinium contrast has become the chosen modality for evaluation of the ulnar side of the wrist, with several prior studies demonstrating improved diagnostic ability compared routine 2D MRI [22–27] and previously supplanting conventional arthrography due to conventional arthrography’s low specificity and accuracy [28].

Given the shape and complex geometry of the TFCC it had been proposed as a candidate for 3D imaging [29]. Additional studies using 3D images of the wrist have been done including a study in healthy volunteers [9], and a study using 3D T1 sequence examining scapholunate, lunotriquietral, and TFCC tears [30]. These studies also showed improved diagnostic potential with 3D imaging. A consensus among these studies as a factor in improved diagnostic potential using 3D isotropic sequence include ability to construct multi-planar reformatted images along an arbitrary obliquity to follow the TFCC and additional ulnar sided structures (or any ligament). Another reason for improved diagnostic potential is the smaller slice thickness of 3D isotropic sequence (0.35 mm versus 2–3 mm) with subsequently decreased partial volume averaging. With smaller slices there is potential for improved identification of subtle abnormalities of the TFCC and capsular complex, which may not bee seen on 2D sequences. Image blur was not a major factor in image quality of the 3 T images, although it has been implicated in the past as a disadvantage to 3 T imaging using long echo train length FSE sequences [31]. Improvement in the imaging of the TFCC is important for many reasons including defining MR anatomy, patient selection for surgical versus non surgical treatment, pre-operative planning, and ultimately leading to improved patient outcomes.

In our study we evaluated 3 T MR wrist arthrogram studies with attention to ulnar wrist structures including TFCC and ulnar capsular complex and compared the image quality of the 3 T sequences to one another and to that of 1.5 T MR wrist arthrogram, along with quantitative analysis amongst the 3 T images using relative SI and relative contrast. The results showed 3 T image quality to be significantly superior to that of 1.5 T and that the 3D isotropic PDFS sequence had the highest scores for image quality among the 3 T sequences. Inter-rater reliability was high for 3 T 3D sequence. In addition, as previous studies, the capability of multi-planar reformations in any obliquity planes proved usefulness for 3D isotropic MR arthrograms at 3 T for improved visualization of TFCC and capsular injuries, especially at the ulnar styloid attachment.

As part of the qualitative grading we looked at the presence of contrast leakage outside the joint capsule at the ulnar wrist, and graded it on confidence of leak and whether it was major or minor, because contrast leakage is frequently seen and is a specific finding with MR arthrogram. The clinical significance of leak at the ulnar side of the wrist, and furthermore whether major or minor plays a role, remains to be seen and is a source of further investigation. Prior studies characterizing leakage on conventional arthrography identified leakage at the ulnar capsular complex as pathologic, supported by improvement in symptoms following repair [32]. However, it has also been found that TFC “communicating defects” on conventional arthrography happen frequently in asymptomatic patients and have also been shown in a significant percentage of cadaveric studies and may in fact represent changes related to degeneration [33–36]. This discussion of contrast extravasation at the ulnar side of the wrist is similar to that found with MR arthrogram of the shoulder, where capsular leakage is also not uncommon, having both pathologic causes including a number of labral tears and conditions such as adhesive capsulitis, but also identified through the subscapular recess along subscapularis and adjacent to axillary recess, both unrelated to needle path and without adjacent related pathologic findings [37]. Regardless, future studies with focus on clinical outcomes will be needed to clarify the significance of contrast material outside the capsule at the ulnar side of the wrist on MR arthrography.

Limitations of our study include the relatively small size, with 11 3 T studies and 10 of which included 3D isotropic sequence. Additionally, the 1.5 T studies were collected over a number of years and the imaging protocols had changed, leaving comparison with similar but not identical imaging parameters for both 3 T and 1.5 T studies. Another limitation is related to the relative longer acquisition time of 3D sequence, which adds approximately 5–6 min to total scan time. The ability to quickly and seamlessly create MPR images is also a limitation, usually requiring additional functional PACS capabilities or additional software, which may not be widely available. The fact that this 3D sequence is PD weighted rather than T1, differentiation between subtle edema and minor contrast leakage occasionally can be difficult. This may suggest 3D isotropic PDFS could potentially replace 2D PDFS, but not 2D T1FS. Finally, selection bias also plays a role given that the majority of patient’s had chronic ulnar wrist pain and had been evaluated by a hand surgeon prior to imaging and initially referred for MR arthrography.

## Conclusion

The results of our initial study investigating the image quality of 3 T MR arthrogram with 3D isotropic PDFS sequence adds to some existing evidence that 3D isotropic PDFS sequences may be useful when imaging the wrist at 3 T. Given the inherent advantages of direct MR arthrography combined with isotropic high-resolution images, this technique shows promise for the evaluation of ulnar wrist pathology including TFCC and capsular injuries.
